# Characterization of the Lassa virus GP1 ectodomain shedding: implications for improved diagnostic platforms

**DOI:** 10.1186/1743-422X-6-147

**Published:** 2009-09-24

**Authors:** Luis M Branco, Robert F Garry

**Affiliations:** 1 Tulane University Health Sciences Center, New Orleans, LA, USA; 2 Autoimmune Technologies, LLC, New Orleans, LA, USA

## Abstract

**Background:**

There is a significant requirement for the development and acquisition of reagents that will facilitate effective diagnosis, treatment, and prevention of Lassa fever. In this regard, detection of early markers of Lassa virus (LASV) infection may improve diagnosis and ultimately successful treatment with antivirals. Characterization of LASV GP1 ectodomain shedding is an important step toward developing sensitive diagnostics to detect circulating levels of this viral glycoprotein in infected patient sera.

**Results:**

Secretion of GP1 from mammalian cells expressing a native LASV GPC gene was not mediated by proteolytic cleavage, as determined by treatment with a panel of matrix metalloprotease (MMP) inhibitors. The shedding of GP1 was also not the result of over-expression of GPC under the control of a strong intron-A containing CMV promoter, as the soluble component could be immunoprecipitated from supernatants of cells expressing low levels of GPC under the control of an intronless promoter. Cells transfected with GPC retained surface membrane-associated expression of GP1 as determined by immunofluorescence assay, in addition to secreting the glycoprotein.

Secreted GP1 derived from GPC expression has a higher content of high mannose N-linked glycosylation than sGP1 expressed independently from the GP2 portion of the protein. Neither GP1 isoform contains sialylated N-glycans, O-linked carbohydrate chains, or galactose-β(1-4)-N-acetylglucosamine commonly present in complex and hybrid N-glycan structures.

**Conclusion:**

These results demonstrate the non-proteolytic secretory nature of GP1 shedding during expression of the arenaviral glycoprotein complex. This phenomenon parallels shedding of a secretory glycoprotein component in filovirus replication. The glycosylation pattern of soluble GP1 resulting from expression of GPC was different from that of a soluble GP1 construct (sGP1-RRAA-FLAG), highlighting the intricately orchestrated post translational processing of the LASV glycoprotein complex.

## Background

Lassa virus, a member of the *Arenaviridae *family, is the etiologic agent of Lassa fever, which is an acute and often fatal illness endemic to West Africa. There are an estimated 300,000 - 500,000 cases of Lassa fever each year [1-3], with a mortality rate of 15%-20% for hospitalized patients and as high as 50% during epidemics [4,5]. Presently, there is no licensed vaccine or immunotherapy available for preventing or treating this disease. Although the antiviral drug ribavirin is beneficial, it must be administered at an early stage of infection to successfully alter disease outcome, thereby limiting its utility [6]. Furthermore, there is no commercially available Lassa fever diagnostic assay, thus preventing early detection and rapid implementation of existing treatment regimens (e.g. ribavirin administration). The lack of adequate countermeasures and means of detection, coupled with the severity of disease, contributed to the classification of LASV as a National Institutes of Allergy and Infectious Diseases (NIAID) Category A pathogen and biosafety level-4 (BSL-4) agent.

The LASV genome is comprised of two ambisense, single-stranded RNA molecules, designated small (S) and large (L) [7]. Two genes on the S segment encode the nucleoprotein (NP) and two envelope glycoproteins (GP1 and GP2); whereas, the L segment encodes the viral polymerase (L protein) and RING finger Z matrix protein. GP1 and GP2 subunits result from post-translational cleavage of a precursor glycoprotein (GPC) by the protease SKI-1/S1P [8]. GP1 serves a putative role in receptor binding, while GP2 has the structural features characteristic of class I viral fusion proteins [9].

Recently we reported that expression of wild type LASV GPC in mammalian cells results in the generation of significant levels of soluble GP1 in the supernatants of transfected cells, that is not associated with GP2 [11]. GP1 ectodomain shedding from cells expressing wild type LASV GPC establishes potential new correlates of disease progression and highlights additional opportunities for development of diagnostics targeting the early stages of Lassa fever. In these studies the mechanism of LASV GP1 ectodomain shedding was further elucidated and its characteristics compared and contrasted to a similar phenomenon in filoviruses, as previously reported for Ebola virus (EBOV) [12].

## Results

### Matrix metalloprotease inhibitors do not affect the secretion of GP1 from LASV GPC expressing cells

**Figure 1:**
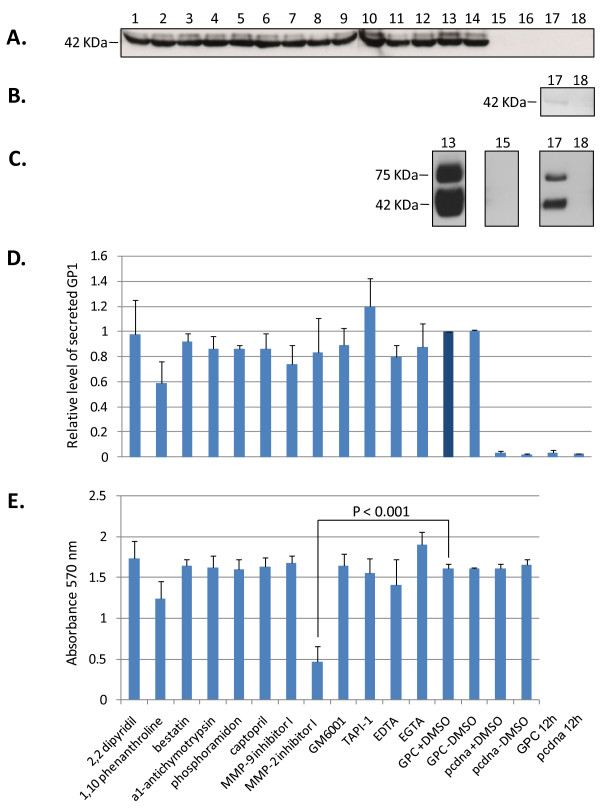
**Effect of metalloprotease inhibitors on the expression and secretion of GP1, and cytotoxicity on LASV GPC-expressing HEK-293T/17 cells**. Cells were transfected for 12 hours, followed by addition of metalloprotease inhibitors. (A) Supernatants were collected at 42 - 45 hours post transfection, cleared by centrifugation, and equal volumes from each reaction were resolved on SDS-PAGE, blotted, and probed with anti-GP1 mAb L52-74-7A. (B) GP1 secretion was detected by western blot analysis as early as 12 hours post transfection, as shown in a longer exposure (lane 17B). Control pcDNA vector was also overexposed in the same blot (lane 18B). (C) Intracellular expression of GPC at the time of harvest (lane 13C), and the corresponding pcDNA vector control (lane 15C), were compared with the same conditions at 12 hours post transfection (lanes 17C, 18C). (D) Relative levels of secreted GP1 were determined from triplicate experiments by densitometry scanning of exposed x-ray films. Data was normalized against GPC+DMSO (= 1) and plotted as mean ± SD, N = 3. Data analysis using ANOVA did not reveal statistically significant differences between any of the conditions containing metalloprotease inhibitors and untreated GPC expressing controls (p > 0.05). (E) MTT cytotoxicity assay from triplicate experiments, plotted as mean A570 nm ± SD, N = 3. ANOVA established a statistically very significant difference (p < 0.001) between MMP-2 inhibitor I and the GPC+DMSO control only. The metalloprotease inhibitors and experimental designations outlined on the x-axis in E. apply to D., and correspond to the numeration in A., B, and C. Positions for secreted and intracellular GP1 (42 KDa), and unprocessed GPC (75 KDa) in cell extracts are noted.

The effects of a diverse set of MMP inhibitors on the secretion of a soluble GP1 component from high level expression of LASV GPC in human cells were investigated. None of the inhibitors employed in these studies resulted in statistically significant reduction in the levels of secreted GP1 from HEK-293T/17 cells expressing LASV GPC (Figure 1A, D, lanes 1 - 12), when compared to untreated controls (Figure 1A, D, lanes 13, 14) [p > 0.05, N = 3]. Relative levels of secreted GP1 for each condition were normalized on GPC expression in the presence of 1% DMSO, the solvent for the majority of inhibitors employed in these studies (Figure 1A, D, lane 13). Inhibitors were added to cells 12 hours after transfection, the earliest time that GP1 could be detected in cell culture supernatants, thereby marking the onset of glycoprotein secretion (Figure 1B, lane 17). The effects of inhibitors on cellular metabolism were measured by reduction of tetrazolium salt (MTT) and quantitated by A570 absorbance in 96 well plates mimicking the conditions used in the 6 well plate format. With the exception of MMP-2 inhibitor 1 (Figure 1E, lane 8) [p < 0.001, N = 3] cellular metabolism was not adversely affected by MMP inhibitors and conditions used in these assays. Despite significantly lowered MTT reduction in cells expressing GPC and treated with MMP-2 inhibitor I, the levels of secreted GP1 were equivalent to those in untreated and control treated cells (Figure 1D, lane 8). Expression of GPC in HEK-293T/17 cells did not adversely affect cellular metabolism, as MTT reduction levels were nearly identical to those obtained with pcDNA vector alone (Figure 1E, lanes 13 - 16).

### Secretory pathway inhibitors abrogate secretion of GP1 from LASV GPC expressing cells

**Figure 2:**
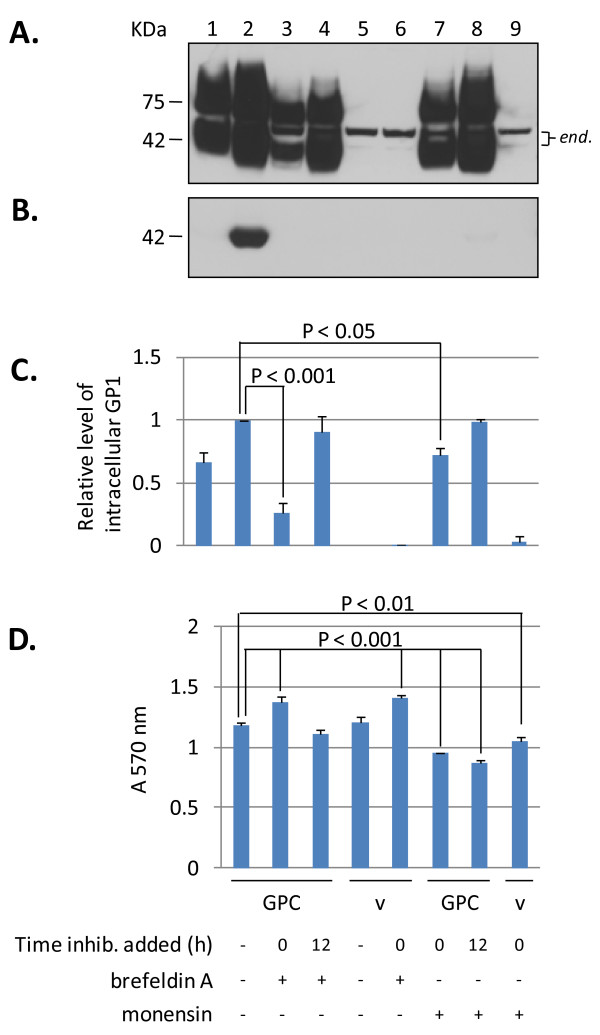
**Inhibition of GP1 secretion from HEK-293T/17 cells expressing LASV GPC by brefeldin A and monensin**. (A) GPC expression in transfected cell extracts. GPC expression was analyzed at 12 (lane 1) and 36 hours (lane 2) post transfection in the absence of inhibitors. Intracellular GPC levels in BFA treated cells at the onset (lane 3), or 12 hours post transfection (lane 4), were compared to monensin treatment at the same time points (lanes 7 and 8, respectively). Vector controls, untreated (lane 5), treated with BFA (lane 6), or monensin (lane 9) only show detection of two endogenous cell proteins (*end.*). (B) GP1 secretion in corresponding culture supernatants. Significant levels of GP1 were detected only in supernatants of GPC transfected, untreated cells (lane 2), although a minor GP1 band was present in monensin treated cell supernatants at 12 hours post transfection (lane 8). (C) Relative levels of secreted glycoprotein in inhibitor treated conditions were compared to untreated GPC expressed sGP1 at 36 hours (= 1). BFA had a more pronounced effect on GPC and GP1 levels when added at the onset (p < 0.001) (lane 3) than at 12 hours post transfection (lane 4), and resulted in lower levels of expressed glycoprotein than under similar conditions with monensin (lanes 7 [p < 0.05] and 8, respectively). (D) MTT assay, plotted as mean ± SD, N = 3. ANOVA established statistically significant differences (p < 0.01 - 0.001) among several conditions when compared to untreated GPC and vector controls. Inhibitors and time of addition to reactions are outlined below D., and apply to all panels. Lane designations above A. apply to all panels.

The effects of secretory pathway inhibitors brefeldin-A (BFA) and monensin on expression of GPC and secretion of soluble GP1 were investigated. At 12 hours post transfection intracellular levels of GPC and processed GP1 were approximately 70% of those at 36 hours (Figure 2A, lanes 1 and 2), although secreted GP1 was barely detectable in cell culture supernatants (Figure 2B, lanes 1 versus 2). Addition of BFA at the onset of transfection with GPC resulted in an average of 80% reduction in intracellular levels of GP1, with undetectable secreted glycoprotein at 36 hours (Figure 2A, B, lane 3). Addition of BFA at 12 hours post transfection resulted in higher levels of accumulated intracellular GP1 than when the inhibitor was added at the onset, but still resulted in undetectable glycoprotein secreted into the culture supernatant. Treatment of cell with monensin resulted in overall higher levels of accumulated intracellular protein than in BFA-treated reactions, both when it was added at the onset or 12 hours post transfection (Figure 2A, lanes 7 and 8). Secretion of GP1 was abrogated when monensin was added at the onset of transfection, although very low levels of the glycoprotein could be detected in supernatants of cells treated with the inhibitor at 12 hours (Figure 2B, lane 8). Overall, treatment with BFA resulted in lowered intracellular GPC expression than with monensin, irrespective of the time of inhibitor addition to culture medium, as assessed by levels of detectable GP1 protein at 36 hours. Inhibition with BFA targets glycoprotein transport within the ER - *cis *Golgi environment, whereas monensin targets transport within the *trans *Golgi apparatus. Thus, BFA is known to affect glycoprotein transport more profoundly in mammalian cells than monensin.

### Secretion of GP1 occurs in cells expressing high or low levels of LASV GPC

**Figure 3:**
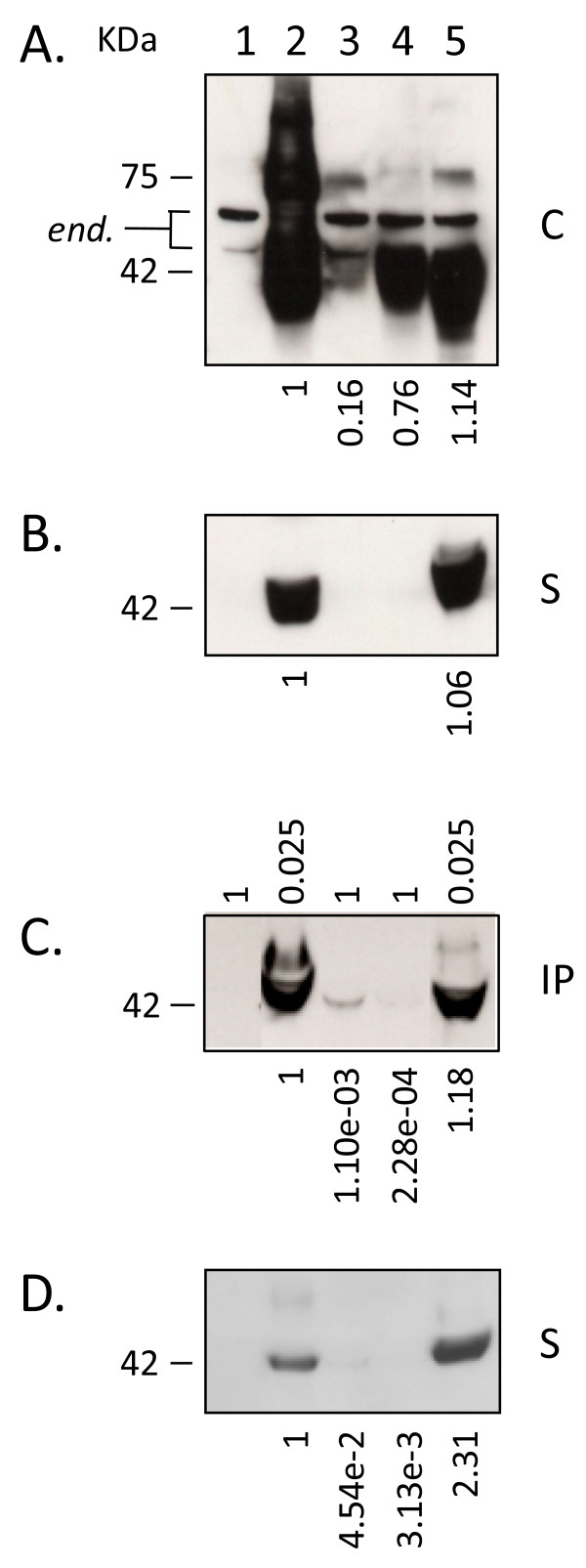
**Intracellular expression and secretion of GP1 in cells transfected with intron-A containing and intronless LASV GPC constructs**. (A) Intracellular expression of glycoprotein constructs [C]. The 42 KDa GP1 protein was readily detected in intron-containing GPC (lane 2), GP1-TM (lane 4), and sGP1-RRAA-FLAG (lane 5) cell extracts. Expression of GPC from an intronless construct resulted in significantly lower levels of detectable GP1 (lane 3). In vector control (lane 1) only endogenous proteins ca. 47 and 55 KDa were detected (*end.*). Relative average expression levels of each glycoprotein compared to intron-A containing GPC are indicated below the respective lanes, as estimated by densitometry. (B) Secreted GP1 was detected only in intron-A containing GPC and sGP1-RRAA-FLAG supernatants [S]. Relative level of secreted GP1 generated by sGP1-RRAA-FLAG compared to GPC, as estimated by densitometry, is indicated below lane 5B. (C) Immunoprecipitation of GP1 from GPC, GP1-TM, and sGP1-RRAA-FLAG transfected cell supernatants [IP]. Immunoprecipitated GP1 was detected in intron-A containing GPC (lane 2C) and sGP1-RRAA-FLAG (lane 5C), and at significantly lower levels in intronless GPC (lane 3C) expressing cell supernatants. A very faint GP1 band was detected in the immunoprecipitated GP1-TM sample (lane 4C). Vector control reactions did not show reactivity (lane 1C). Relative volume of each IP reaction loaded per lane is indicated above panel C. (D) Secreted GP1 profile in VERO cells transfected with LASV glycoprotein expression constructs. Average levels of GP1 from intronless GPC, GP1-TM, and sGP1-RRAA-FLAG, compared to those from intron-A containing GPC expression are shown below each lane in panels C and D (N = 4).

To assess if secretion of LASV glycoprotein is a phenomenon resulting from high level expression of GPC, intracellular and secreted GP1 levels driven by intron-A containing or intronless CMV promoter constructs were analyzed and quantitated. In HEK-293T/17 cells the average level of intracellular GP1 expression derived from intronless CMV promoter constructs was 16% of that obtained with intron-A counterparts (Figure 3A, lane 3). Expression from the weaker CMV promoter resulted in undetectable levels of secreted GP1, as assessed by western blot analysis of cell culture supernatants (Figure 3B, lane 3). In an attempt to detect secreted GP1 from lower intracellular GPC expression, LASV GP1-specific mAb L52-74-7A was used to immunoprecipitate (IP) the glycoprotein from cell culture supernatants. One millilitre of each supernatant was subjected to IP with anti-GP1 mAb and Protein G Sepharose, and the resulting immunoprecipitates were detected by western blot. As shown in Figure 3C the entire IP reaction (1) was resolved for control vector (lane 1), intronless GPC (lane 3), and control GP1-TM (lane 4) constructs. Conversely, a fraction (0.025) of each of the intron-A driven GPC and sGP1 constructs were analyzed in the same blots for comparison (Figure 3C, lanes 2 and 5). The intracellular ratios of GP1 between intron-A driven and intronless GPC constructs were calculated linearly based upon densitometry. Whereas intracellular GP1 levels derived from expression of an intronless GPC construct were, on average, 16% of those obtained with the intron-A containing counterpart, the resulting secreted GP1 was only 0.11%, or ~1/100 of the cellular ratio (Figure 3C, lane 3). A very minor GP1 band could be immunoprecipitated from the supernatants of GP1-TM expressing cells (Figure 3C, lane 4). The GP1-TM construct contains the LASV GP2 TM domain directly fused to the C-terminus of the GP1 ORF, and the SKI-1/S1P protease cleavage site, RRLL, remains intact. In this conformation the vast majority of GP1-TM is not cleaved, and GP1 is produced almost exclusively as a membrane-anchored form. Expression of LASV glycoprotein constructs in VERO cells generally produced similar results as those obtained with HEK-293T/17 cells (Figure 3D), with the notable exception of higher secreted levels of GP1 from intronless CMV promoter-driven GPC (Figure 3D, lane 3). Also, secreted levels of GP1 from a sGP1_RRAA-FLAG construct were approximately twice those observed with intron-A containing GPC (Figure 3D, lane 2 versus lane 5). In contrast, the same corresponding levels in HEK-293T/17 cells were nearly matched (Figure 3A, C, lane 5). To further assess the levels of secreted GP1 from VERO cells expressing GPC under the control of an intronless CMV promoter, supernatants were subjected with IP with mAb L52-74-7A. Western blot analysis revealed that significantly higher levels of sGP1 could be purified from the supernatants of VERO cell expressed intronless GPC than from HEK-293T/17 cells, as expected, based on analysis on non-immunoprecipitated samples (data not shown).

### GP1 remains associated with the cell membrane in LASV GPC expressing cells

**Figure 4:**
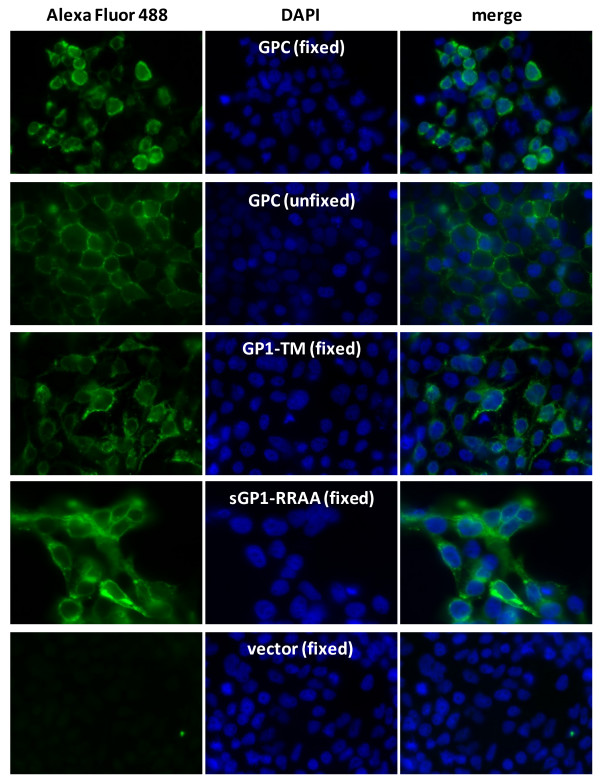
**Expression profile of LASV GPC, GP1-TM, and sGP1-RRAA-FLAG in HEK-293T/17 cells by immunofluorescent microscopy**. HEK-293T/17 cells grown on cover slips and transfected with LASV glycoprotein constructs were stained with anti-GP1 mAb L52-74-7A and Alexa Fluor 488 secondary antibody, and counterstained with DAPI. Cells were fixed and permeabilized prior to staining (panels A, C, D, and E), or were stained live (panel B). Each panel shows captured fluorescent images of cells at 60× magnification, in oil immersion, with the same exposure settings used throughout for the given fluorochrome (300 ms for FITC channel; 175 ms for DAPI channel). Cell surface and intracellular GP1 (Alexa Fluor 488, panels A - E), stained nuclei (DAPI, panels A - E), and merged images (merge, panels A - E) were captured with Slidebook 5.0 software (Intelligent Imaging Innovations, Inc., Denver, CO). Images were not deconvolved or processed further. Each image is representative of at least 20 fields photographed and compared for each experimental condition.

Expression and localization of LASV GP1 in HEK-293T/17 cells was analyzed by immunofluorescence assay (IFA) with mAb L52-74-7A on fixed and permeabilized, or live, unfixed cells (Figure 4). All constructs used in LASV glycoprotein expression for IFA were driven by intron-A containing CMV promoters. Expression of GPC on fixed cells was generally diffuse throughout the cytoplasm, with bright distribution of the antigen throughout the peripheral outline of stained cells (Figure 4A, panels 1 and 3). Expression of GPC on live cells, which were not permeable to the antibody, and thus stained antigen present of the outer leaflet of the membrane, showed primarily bright punctate staining throughout the cellular periphery (Figure 4B, panels 1 and 3). Unfixed, GPC-expressing cells were extensively washed with staining buffer prior to incubating with GP1-specific mAb to remove soluble GP1 from the reactions. Thus, staining with mAb L52-74-7A on GPC-expressing unfixed cells detected GP1 associated with the outer leaflet of the cell membrane. GP1-TM expression on the cell surface was largely similar to that observed with GPC on unfixed cells, with additional diffuse stained throughout the cytoplasm (Figure 4C, panels 1 and 3). Soluble GP1 expression from sGP1-RRAA-FLAG displayed diffuse intracellular staining, in addition to bright antigen distribution throughout the periphery of transfected cells (Figure 4D, panels 1 and 3). The LASV GP1-specific mAb L52-74-7A did not stain empty vector transfected cells (Figure 4D, panels 1 and 3). Cells were counterstained with DAPI to enumerate nuclei (Figure 4A-D, panels 2 and 3).

### Secreted GP1 derived from expression of LASV GPC and sGP1 have disparate glycosylation patterns

**Figure 5:**
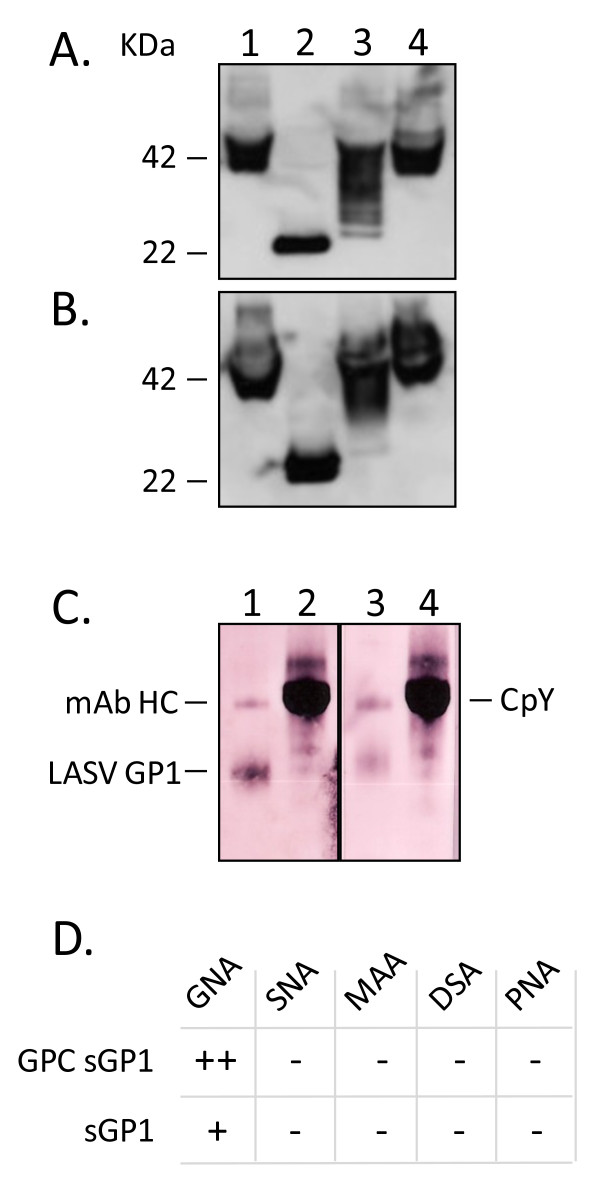
**Glycosylation profile of GPC and sGP1-RRAA-FLAG-derived secreted GP1 assessed by digestion with endo- and exo-glycosidases, and by lectin binding profiles**. Immunoprecipitated secreted GP1 from LASV GPC expression (A) or sGP1-RRAA-FLAG expression (B) was subjected to digestion with PNGase F (lane 2), Endo H (lane 3), or neuraminidase (lane 4). Untreated glycoprotein controls were similarly processed, as outlined in methods, but enzymes were not added to reactions (lane 1). Proteins were subjected to SDS-PAGE and western blot analysis with biotinylated anti-GP1 mAb L52-74-7A and SA-HRP. Molecular weights of fully glycosylated GP1 (42 KDa) and the completely deglycosylated form of the protein (22 KDa), are indicated. (C) GNA lectin binding profile on immunoprecipitated GP1 from GPC expression (lane 1) or sGP1-RRAA-FLAG (lane 3). Control GNA binding for each reaction was performed on CpY (lanes 2 and 4). The heavy chain of mAb L52-74-7A reacted with GNA, and is indicated (mAb HC). Secreted GP1 from GPC expression reacted significantly stronger with GNA (lane 1) than with sGP1-RRAA-FLAG (lane 3). (D) Complete lectin panel binding profile on secreted GP1 from GPC or sGP1-RRAA-FLAG.

The glycosylation profile of secreted GP1 was derived by treatment of immunoprecipitated protein with PNGase-F, Endo-H, or Neuraminidase, followed by western blot analysis of protein migration on SDS-PAGE. The LASV GP1 glycoprotein contains 7 predicted N-linked glycosylation sites that could be accessible to removal by PNGase-F. Secreted GP1 from GPC expression was fully deglycosylated by PNGase-F, as determined by a loss of ca. 20 KDa from the mass of the protein detected on western blots (Figure 5A, lane 2). The expected mass of LASV GP1 without carbohydrate moieties is 22771.82 Daltons (ExPASy Compute pI/Mw tool [13-15]). Expression of sGP1-RRAA-FLAG produced a secreted form of GP1 that was largely deglycosylated by PNGase-F, with an additional moiety at ca. 24 KDa that could not be further deglycosylated with higher levels of enzyme, additional digestion time, or further denaturation (N = 4) (Figure 5B, lane 2). Digestion of secreted GP1 from GPC expression with Endo H produced a highly heterogeneous banding pattern on western blots, with moieties ranging from the fully glycosylated 42 KDa protein to approximately 25 KDa (N = 4) (Figure 5A, lane 3). Conversely, sGP1-RRAA-FLAG was less susceptible to Endo H digestion, producing mainly a heterogeneous banding pattern between 42 KDa and 35 KDa, with an additional faint band at ca. 25 KDa (N = 4) (Figure 5B, lane 3). Neither form of the secreted GP1 protein resulted in mobility shifts upon digestion with Neuraminidase (N = 3) (Figure 5A, B, lane 4). The carbohydrate composition of both GP1 forms was further dissected with a panel of lectin binding assays. Among the five lectins employed, each with well characterized carbohydrate binding specificities, only *Galanthus nivalis *agglutinin (GNA) bound to LASV GP1, albeit differentially. The lectin bound significantly stronger to GPC-derived sGP1 (Figure 5C, lane 1) than to sGP1-RRAA-FLAG (Figure 5C, lane 3) (N = 3). The amount of glycoprotein loaded per lane was assessed by densitometry analysis of GP1 detected on western blots with mAb L52-74-7A, and determined to be nearly equal in every experiment (data not shown). The internal kit control protein for GNA binding, carboxyl peptidase Y (CpY), was run alongside LASV GP1 and stained equivalently in both assays (Figure 5C, lanes 2 and 4).

### Secreted GP1 from GPC and sGP1 expression have identical N-terminal sequences

Immunoprecipitated and PNGase-F deglycosylated GP1 protein from GPC and sGP1-RRAA-FLAG were subjected to SDS-PAGE and transferred to PVDF membranes for N-terminal sequence analysis by Edman degradation. The six N-terminal amino acids from each protein were T-S-L-Y-K-G, thus representing the SPase-cleaved form of the LASV GP1 protein, as previously characterized [8].

## Discussion

A panel of soluble MMP inhibitors with a wide range of specificities was employed in the characterization of protease-mediated GP1 shedding from mammalian cells expressing LASV GPC. A broad spectrum hydroxamic acid inhibitor of MMPs, GM 6001 (inhibits MMP1, 2, 3, 8, 9), metal ion chelators (EDTA, EGTA, 2,2 dipyridyl), a specific inhibitor of chymotrypsin-like serine proteases (α_1_-antichymotrypsin), a cell surface aminopeptidase inhibitor (Bestatin), an inhibitor of thermolysin-like protease and metalloendopeptidases (Phosphoramidon), a broad MMP inhibitor (phenanthroline), TNF-α convertase (TACE) protease inhibitor-1 (TAPI-1), and specific inhibitors of MMPs (MMP2, 9), were added to growth medium of LASV GPC-expressing mammalian cells in an attempt to determine their role in the release of soluble GP1. None of the inhibitors showed statistically significant inhibition of GP1 shedding from HEK-293T/17 cells expressing LASV GPC, although most compounds were tested at concentrations significantly greater than reported IC_50 _values (Table 1). These results strongly suggest that GP1 is released from cells as a soluble and uncoupled protein arising from the intracellular cleavage and processing of the GPC complex. Further, it can be proposed that the 1:1 stoichiometry of GP1 and GP2 in cells expressing GPC does not result in a 1:1 ratio of assembled tripartite complex on the cell surface. It is possible that tripartite complex assembly in the ER results in retention of a fraction of GP2 in favour of GP1 secretion. This model is partly supported by our previous studies that established a critical role for GP1 as a chaperone for correct processing and secretion of GP2 [11]. These studies also suggested that cell surface expression of GP2 without associated GP1 was unlikely; GP2 expression resulted in very low levels of intracellular protein, and it was undetectable on the cell surface (unpublished data). Cytotoxicity of MMP inhibitors was monitored by MTT uptake assays. MMP-2 inhibitor I resulted in significantly cytotoxicity compared to untreated controls (p < 0.001, N = 3), but the resulting release of GP1 from cells was not significantly affected (Figure 1A, D, E, lane 8), indicating that *de novo *protein synthesis and cellular secretory pathways were not targeted by the inhibitor.

**Table 1:** Effects of matrix metalloprotease and secretory pathway inhibitors on GP1 shedding and cytotoxicty in HEK-293T/17 cells

**inhibitor**	**[] in assay**	**Notes/references**	**effect on GP1 shedding**	**cytotox.(MTT)**	**morphology (microscopy)**
2,2 dipyridyl	1 μM	Calbiochem	-	-	enlarged cells

1,10 phenanthroline	10 μM	ref. [12]	-	-	detached cells

bestatin	10 μM	1 - 10 μM working []	-	-	enlarged cells

α1-antichymotrypsin	4 μM	ref. [12]	-	-	normal

phosphoramidon	10 μM	IC_50 _= 4.6 μM	-	-	enlarged cells

captopril	10 μM	ref. [12]	-	-	normal

MMP-9 inhibitor I	50 nM	IC_50 _= 5 nM	-	-	detached cells

MMP-2 inhibitor I	100 μM	K_i _= 1.7 μM	-	+	enlarged cells

GM6001	5 μM	K_i _= 400 pM - 27 nM	-	-	normal

TAPI-1	50 μM	IC_50 _= 100 nM	-	-	normal

EDTA	5 mM	1 - 10 mM working []	-	-	rounded cells

EGTA	5 mM	1 - 10 mM working []	-	-	rounded cells

brefeldin A	15 μg/mL	BD Golgi Plug™	+++	+	rounded cells

monensin	10 μM	BD Golgi Stop™	+++	+	rounded cells

The mammalian expression system employed in these studies was originally developed for high level production of recombinant LASV proteins for diagnostic assay development [11]. High level expression of soluble LASV GPC resulted in both secretion of glycoprotein precursor uncleaved by SKI-1/S1P, and high levels of sGP1. Although CMV promoter strength has not been compared to that of the LASV promoter, it is reasonable to suggest that very high level expression of recombinant LASV genes in mammalian cells could result in artefactual processing, secretion, and stoichiometry of glycoprotein complex components. It is evident that high level expression of soluble GPC in HEK-293T/17 cells driven by CMV plus intron-A overwhelms cellular SKI-1/S1P protease, as approximately one half of the secreted glycoprotein is uncleaved [11]. In order to address the possibility that overexpression of GPC resulted in artefactual secretion of GP1 we expressed the glycoprotein from an intronless CMV-driven promoter. Expression of GPC from CMV-intron-A driven vectors resulted in 8 - 10 fold higher levels of cleaved intracellular GP1 than from intronless counterparts. Low levels of sGP1 in the supernatants of cells expressing intronless GPC were detected only when the glycoprotein was immunoprecipitated. An 8 - 10 fold dilution of culture supernatant from cells expressing sGP1 still resulted in detection of significant levels of secreted glycoprotein, whereas similar levels of immunoprecipitated GP1 from intronless GPC expression required 400 fold higher volume of supernatant. These results suggest that overexpression of GPC results in disproportionately enhanced secretion of sGP1 when compared to lower expression levels of the glycoprotein. Generally, expression and secretion levels of GP1 in the HEK-293T/17 line paralleled those obtained with VERO cells, a common line used for the propagation of Lassa virions and analysis of LASV gene expression (Figure 3D).

Association of GP1 with the cell membrane as a component of the tripartite glycoprotein complex was verified by staining live cells overexpressing GPC. After extensive cell washing to remove sGP1, followed by staining with a LASV GP1-specific mAb, cell surface associated GP1 could be readily detected. The staining pattern was less diffuse than with fixed and permeabilized cells overexpressing GPC, with bright fluorescence primarily associated with the cell membrane. Thus, a fraction of the GP1 protein associates with GP2 and SSP on the cell membrane of GPC-transfected cells, whereas a soluble, un-associated component is shed into the cellular milieu.

Sequencing of GP1 protein from expression of GPC or sGP1 revealed identical N-termini through six amino acids, suggesting that both forms of the glycoprotein result from cleavage of pre-GPC by SPase at amino acid 58, and that the SSP functions as the secretory signal.

Protein deglycosylation and lectin binding studies revealed significant heterogeneity in the carbohydrate composition of the LASV GP1 protein. Whereas secreted GP1 from expression of LASV GPC could be fully deglycosylated with PNGase F (Figure 5A, lane 2), two distinct forms could be observed in sGP1 from sGP1-RRAA-FLAG expression. The 22 KDa band corresponding to a fully deglycosylated sGP1 migrated just below a ca. 24 KDa form of the protein, and it was consistently detected on western blots (Figure 5B, lane 2). The average 14-residue precursor of N-linked oligosaccharides adds approximately 2.6 KDa to the mass of a target protein ([GlnNAc × 3] + [Man × 9] + [Glc × 3]). Thus, the 24 KDa form of sGP1-RRAA-FLAG that remains after digestion with PNGase F suggests the presence of an oligosaccharide branch other than N-linked in nature. Alternately, as PNGase F is not able to cleave N-linked glycans from glycoproteins when the innermost GlcNAc residue is linked to an α1-3 Fucose residue [16], the possibility of this form of linkage in sGP1-RRAA-FLAG exists. This type of modification is most commonly found in plant and some insect glycoproteins, however. Computational analysis of the LASV GP1 protein sequence using OGPET v1.0 (University of Texas, El Paso [17] revealed the possibility of an O-GalNAc (mucin type) glycosylation in GP1 at Ser 80 (SnI**S**TnHL), with a predictive score of 0.599833 (cutoff = 0.4247). Additionally, NetGlycate 1.0 Server [18], a predictor of glycation of ε amino groups of lysines in mammalian glycoproteins, attributed a score of 0.914 to Lys5 in LASV GP1, which is significantly above the threshold of 0.5. Lectin-binding assays did not aid in resolving the glycosylation pattern of the 24 KDa form of sGP1-RRAA-FLAG. The lectins PNA (Peanut agglutinin) and DSA (*Datura stramonium *agglutinin), which recognize the core disaccharide galactose (1-3) N-acetylgalactosamine, and Gal-(1-4)GlcNAc in complex and hybrid glycans, in O-glycans, and GlnNAc in O-glycans, respectively, did not bind to the 24 KDa sGP1 form. A higher degree of N-linked glycosylation heterogeneity on LASV GP1 was obtained in Endo H digestion profiles. Endo H removes only high mannose and some hybrid types of N-linked carbohydrates from glycoproteins, and will not cleave more complex glycan structures [19,20]. Soluble GP1 from GPC expression revealed a more heterogeneous distribution of high mannose and hybrid glycosylation than were present in sGP1-RRAA-FLAG, indicating a higher content of mannose and/or hybrid structures. Conversely, it appears that sGP1-RRAA-FLAG has a significantly higher content of complex glycan structures, as determined by reduced EndoH activity on the glycoprotein. These results were confirmed by the differential staining of both GP1 forms by GNA; soluble GP1 from GPC expression was heavily stained with GNA, whereas sGP1-RRAA-FLAG showed significantly dimmer reactivity. Densitometry analysis showed a ratio of 2.27 ± 0.014 in GNA staining intensity of GPC-derived GP1 versus sGP1-RRAA-FLAG (N = 3). The marked differences in glycosylation of each form of LASV GP1 could be attributed to a potentially different translational and post-translational modification profile and timeline in expressing cells. The process of translation, ER translocation, SPase and SKI-1/S1P cleavage, ER retention of glycoproteins, and assembly of the LASV tripartite complex (GP1+GP2+SSP) has been somewhat dissected, and constitutes a finely orchestrated process in the ER and Golgi of mammalian cells. The correct processing and assembly of the tripartite complex is essential for subsequent binding of LASV virions via GP1 to the α-dystroglycan cellular receptor, and initiation of viral entry via the fusion peptide on the N-terminus of GP2, a process largely mediated by the presence of the intact SSP peptide. Thus, it is conceivable that LASV GPC-derived glycoproteins remain accessible to ER resident glucosyltransferases, and the homologous lectins calnexin and calreticulin longer than sGP1-RRAA-FLAG, resulting in an overall different endpoint glycosylation pattern. Glucosyltransferases act as one of the primary protein folding quality control mechanisms in the ER, in concert with lectins, peptidyl-propyl-isomerases, and chaperones that are tightly regulated by nuclear transcription mediated by ER membrane resident proteins (e.g. ATF6) that sense high levels of protein misfolding in this organelle [21]. The formation of hybrid and complex glycans takes place in the *medial *and *trans *Golgi compartments. Therefore, proteins residing in the ER which have not passed through the Golgi cannot possess hybrid or complex N-linked structures. Thus, the intricate assembly process for the LASV GPC complex would be expected to differ from that of single glycoprotein expression from the sGP1-RRAA-FLAG construct. The kinetics of GP1 secretion from expression of GPC and sGP1-RRAA-FLAG do not differ significantly, as the onset for detection of each form of the glycoprotein in the extracellular medium occurs at approximately 12 hours post transfection (with CMV+intronA driven constructs). Post-translational processing and secretion of sGP1-RRAA-FLAG is not necessarily faster than GPC expressed GP1, but it is executed differently. It is noteworthy to point that such findings contrast with previously reported results by our group that showed clear and complete removal of high mannose glycans from sGP1 and sGP2 generated with the same expression constructs and cell type [11]. Such contrasting results point to subtle differences in culture conditions that could significantly affect resulting glycosylation patterns. Although the HEK-293T/17 clone used in both studies was obtained from the same source (American Type Culture Collection [ATCC]), the studies were performed with different lots of culture media and supplements, namely fetal bovine serum. It has been extensively reported that subtle differences in cell culture environments, including changes in medium composition can result in significant differences in glycosylation patterns of secreted glycoproteins. Such impact on glycosylation is a primary focus of product quality control in many glycoprotein-based biopharmaceutical products, such as therapeutic antibodies, erythropoietin, and reproductive hormones [22-26]. Lastly, treatment of secreted GP1 from GPC and sGP1-RRAA-FLAG expression did not result in observable shifts with neuraminidase. Neuraminidase (Acetyl-neuraminyl hydrolase; Sialidase) catalyzes the hydrolysis of α2-3, α2-6, and α2-8 linked N-acetyl-neuraminic acid residues from glycoproteins and oligosaccharides [27,28]. These results were further confirmed by the lack of binding to both forms of GP1 by the lectins MAA (*Maackia amurensis *agglutinin) and SNA (*Sambucus nigra *agglutinin), which are specific for α2-3 and α2-6 galactose, respectively. Thus, it appears that LASV GP1 is not synthesized with terminal sialic acid residues in its carbohydrate profile, irrespective of the glycoprotein precursor.

It is well known that hydrophobic interactions predominate over the proper and improper folding of proteins. Further, the hydrophobic areas of high mannose N-glycans are closely associated with promotive effects on protein folding, possibly depressing the hydrophobic interactions unfavorable to the proper folding of nascent polypeptides in the E.R. It has been established by Jitsuhara et al. [29], Narhi et al. [30], and others that higher oligomannose chains of glycosylated proteins promote protein folding more strongly than lower ones [31]. Of noteworthy mention is the folding of influenza virus hemagglutinin with a truncated N-glycan Glc_1_Man_5_GlcNAc_2 _moiety that normally interacts with the lectin calnexin in the E.R. is markedly depressed [32]. The E.R. resident lectins calnexin and calreticulin tether monoglucosylated high mannose N-glycans from nascent proteins, resulting in suppression of aggregation, thus promoting protein folding [33]. Many studies have established the correlation between proper protein glycosylation with the acquisition and retention of glycoprotein biological activities. It is reasonable to expect that in the arenaviral tripartite complex the proper folding of the individual glycoprotein subunits, and the interaction with the SSP, have critical roles in establishing functional binding of virions to host cell receptors and subsequent fusion and viral entry. Our own previous LASV GP1 protein expression studies in multiple biological systems have resulted in markedly different results. Although expression of full length GP1 in *E. coli *was achievable, the lack of glycosylation in the prokaryotic system required the presence of sodium dodecyl sulfate (SDS), reducing agents (e.g. dithiothreitol [DTT]), and the zwitterion morpholino-ethanesulfonic acid (MES) in the purification scheme in order to yield non-aggregated protein [34]. Further processing of bacterial expressed GP1 protein for analytical assays that required removal of SDS and DTT readily resulted in aggregation. In contrast, expression of multiple forms of LASV GP1 in mammalian cells always resulted in glycosylated, soluble protein, although aggregation of the standalone glycoprotein remained an issue [11]. Further characterization of post-translational processing of the arenaviral glycoprotein complex may be of crucial importance in the establishment of viral infectivity correlates, as well as pave the way for identification of disease susceptibility markers, and development of effective vaccines and therapeutics.

**Figure 6:**
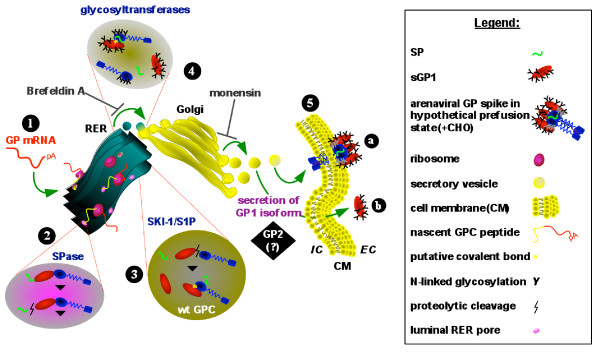
**Schematic representation of arenaviral glycoprotein expression pathway and secretory forms**. Arenaviral glycoprotein (GP) mRNA is transcribed from plasmid DNA in the host cell (1), and is directed to the rough endoplasmic reticulum (RER) for translation by associated ribosomes. The nascent polyprotein is directed to the RER lumen by the unique arenaviral SP, where SPases cleave the SP from the GP polyprotein precursor (2). In the RER the GP precursor is cleaved by SKI-1/S1P to yield GP1 and GP2 subunits (3). Wild type GP is possibly comprised of covalently linked, or non-covalently associated GP1 and GP2 subunits, and associated SP (3). Glycosylation of GP1 and GP2 is mediated by glycosyltransferases in the RER or in the Golgi cisternae (4). Membrane bound vesicles transport proteins to the *cis *face of the Golgi where final processing and assembly of the GP complex occurs. Fully assembled and glycosylated GP complex or subunits are packaged in exocytic vesicles which emerge from the *trans *face of the Golgi and are directed toward the plasma membrane. Glycoprotein complex is anchored to the cell membrane via the transmembrane domain of GP2 (5a). A complexed heterodimeric GP1-GP2 is displayed in 5a. Extracellular GP1 originating from GPC expression is a secreted glycoprotein isoform (5b). The positions of BFA and monensin inhibitory action along the secretory pathway are indicated. The role of LASV GP2 in the secretory pathway of GP1 was not elucidated in these studies (black box). A graphic representation of each relevant component in the outlined pathway is shown in the legend box.

The present studies affirm the transition of GP1 from the ER to the Golgi in its path toward secretion, and that the secretory component of GPC follows the same route. Complete or nearly complete inhibition of GP1 secretion in supernatants of cells transfected with GPC and treated with BFA and monensin is indicative of a secretory pathway mediated phenomenon. This conclusion is further strengthened by the lack of inhibition of GP1 secretion from GPC-expressing cells treated with a wide range of MMP inhibitors. Despite secretion of GP1 from GPC-expressing cells, a surface membrane-associated GP1 component was specifically visualized on live cells. These observations are indicative of a secreted GP1 component, in addition to the presence of a cell surface expressed, functional tripartite glycoprotein complex. A proposed model outlining the secretory pathway of LASV GP1 is graphically displayed in Figure 6.

Together, these data strongly suggest dual modalities in LASV GPC expression; the formation of a fully functional tripartite complex for display on the surface of infectious virions, and the secretion of a soluble GP1 component with as of yet uncharacterized function. The fate of un-associated GP2 during expression of LASV GPC was not investigated in these studies, but it warrants analysis, as the stoichiometry of the glycoprotein complex depends on the ratio of the available individual components. Dolnik et al. [12] have partly characterized the role of a soluble GP component in EBOV, and have proposed a role in the infectivity of the virus *in vivo*. According to Dolnik et al., the secreted EBOV GP component acts as an immune decoy, by targeting the humoral immune system and possibly removing from circulation antibodies with innate neutralizing activity against the virus. This mechanism would provide circulating infectious virions a clearer path toward infection of new target cells, with enhanced virulence. Also, Wahl-Jensen et al. have further delineated the role of secreted EBOV GP in activation of primary target cells [35,36]. These studies revealed that rigid presentation of the EBOV glycoprotein, as it's displayed on the surface of virions is critical for activation of pro-inflammatory cytokines and chemokines, whereas soluble glycoprotein failed to elicit any response. Additionally, EBOV GP(1,2) in viral particle-associated form mediated endothelial cell activation and decreased cell barrier function, whereas sGP, the major soluble glycoprotein of EBOV, demonstrated an anti-inflammatory role by protecting the endothelial cell barrier function.

Although a similar mechanism has not been ascribed to arenaviruses, our work presents a platform upon which to investigate this phenomenon *in vivo*. The secreted GP1 component resulting from GPC expression has not been detected in the serum of LASV infected patients, and further work will be performed by our group in an attempt to test the feasibility of this hypothesis. If the phenomenon of GP1 shedding is present *in vivo *the glycoprotein should be preferentially detected in the early stages of arenaviral infection, when circulating antigens are present at the highest levels. Soluble GP1 in serum could be separated from virions by selective size filtration, thus providing a simple and easily deployable platform for identification of the soluble glycoprotein component. If confirmed, arenaviral glycoprotein shedding could result in the development of more sensitive and earlier detection diagnostics, as well as establish new correlates of pathogenesis and immune evasion by this class of hemorrhagic fever viruses.

## Conclusion

The shedding of GP1 from cells expressing GPC results from secretion of a cleaved and un-associated fraction of the protein. This phenomenon is not the result of cleavage of GP1 from the tripartite complex expressed on the cell surface, and it is not mediated by cellular MMPs. Instead, secretion of GP1 occurs when GPC is processed into GP1 and GP2 in the ER, and a significant fraction of this protein is shuttled through the secretory pathway and is released into the extracellular medium. Both forms of secreted GP1 share N-terminal sequences, but have dissimilar glycosylation profiles that suggest a differential retention and glycoprotein processing timeline in the ER and Golgi compartments.

## Methods

### Cells, plasmids, antibodies

HEK-293T/17 cells (ATCC CRL11268) were maintained in complete high glucose Dulbecco's Modified Eagle Medium (cDMEM) supplemented with non-essential amino acids (NEAA) and 10% heat-inactivated fetal bovine serum (ΔFBS).

Vero cells (ATCC CRL 1587) were maintained in cDMEM supplemented with NEAA and 5% ΔFBS. Plasmid constructs expressing LASV GPC, GP1-TM, and sGP1, and the backbone vector pcDNA3.1+zeo:intA were described elsewhere [11]. For immunoassays, Dr. Randal Schoepp kindly provided the LASV-specific GP1 mAb L52-74-7A, which was generated against purified gamma-irradiated LASV, as previously described [37]. Horseradish peroxidase (HRP)-conjugated secondary antibodies specific for mouse IgG were purchased from Kirkegaard and Perry Laboratories (KPL, Gaithersburg, MD). Alexa Fluor 488-conjugated goat F(ab')_2 _anti-mouse IgG secondary antibodies for immunofluorescence assays were purchased from Molecular Probes. Anti-LASV GP1 mAb L52-74-7A was biotinylated with EZ-Link^® ^NHS-Biotin (Pierce, Rockford, IL), and desalted with Zeba™ Desalt Spin Columns (Thermo Scientific, Rockford, IL), according to manufacturer's instructions.

### Transient expression of LASV gene constructs

Recombinant LASV protein expression was analyzed in HEK-293T/17 or VERO cells transiently transfected with mammalian expression vector DNAs, which were prepared using the PureLink HiPure plasmid filter midiprep kit (Invitrogen, Carlsbad, CA). The negative control vector pcDNA3.1(+):intA was included in all transfections. Briefly, 1 × 10^6 ^cells were seeded per well of a Poly-D-Lysine-coated 6-well plate in 2 mL of cDMEM. After overnight incubation in growth conditions cells were transfected with unrestricted recombinant plasmid DNAs using Lipofectamine™ 2000 (Invitrogen), according to the manufacturer's instructions. Four μg of each plasmid DNA were used per transfection. Transfections were incubated for the times described in results, at 37°C, 5% CO_2_, 90% RH, after which cell culture supernatants were collected and clarified by centrifugation. To prepare cell extracts from transfected cultures, cell monolayers were carefully washed twice with Ca^++^- and Mg^++^-free PBS, pH7.4, collected by gentle dislodging, transferred to 1.5 mL polypropylene tubes, and lysed for 10 minutes in a mammalian cell lysis buffer comprised of 50 mM Tris buffer, pH 7.5, 1 mM EDTA, 0.1% SDS, 0.5% deoxycholic acid, 1% Igepal CA-360, and a protease inhibitor cocktail (Sigma Aldrich, St. Louis, MO), according to the manufacturer's instructions. The insoluble fraction was pelleted by centrifugation at 14,000 × g for 10 minutes, and the supernatants were transferred to fresh tubes. Protein concentration was determined for each sample by A280 with A260 subtraction, and verified using a Micro BCA™ Protein Assay Kit, as outlined by the manufacturer (Thermo Scientific).

### Metalloprotease and secretory pathway inhibition assays

Metalloprotease inhibitors were purchased from Sigma (1,10-Phenanthroline monohydrate, 2,2'-Dipyridyl ReagentPlus^®^, Bestatin hydrochloride, Phosphoramidon disodium salt, α_1_-Antichymotrypsin, EDTA, EGTA) or Calbiochem (MMP-2 Inhibitor I, MMP-9 Inhibitor I, InSolution™ GM 6001, InSolution™ TAPI-1, Captopril), and resuspended in 100% DMSO (Sigma), or with cell culture medium when appropriate, at 100× concentration. Inhibitors were diluted in growth medium for analysis, at a final DMSO concentration of 1%. For inhibition assays HEK-293T/17 cells were transiently transfected with plasmid DNAs in 6-well plates, as described above, for 12 hours prior to the addition of metalloprotease inhibitors. Transfection medium was then removed and cells were washed twice with 1× PBS, pH7.4, followed by addition of 2 mL of fresh growth medium containing inhibitors. Plates were returned to the growth chamber and reactions were harvested between 42 - 45 hours post transfection by collecting supernatants and generating whole cell protein extracts, as described above. The Golgi secretory pathway inhibitors brefeldin A and monensin were purchased from Sigma, and diluted in growth medium to 15 μg/mL and 10 μM, respectively. Inhibitors were added to cells at the time of transfection with pcDNA3.1(+):intA or pcDNA3.1(+):intA:LASV GPC, or at 12 hours. Supernatants and cell extracts were harvested at 36 hours post transfection for western blot analysis.

### Western blot and densitometry analyses

Expression of LASV GP1 in cell extracts and culture supernatants was confirmed by Western blot analysis using anti-LASV GP1 mAb L52-74-7A and a horseradish peroxidase (HRP)-conjugated goat anti-mouse IgG (H+L) secondary antibody. Briefly, 10 μg of total cell protein in 10 μL (~1 × 10^5 ^cell equivalents), or 26 μL of cell culture supernatant were resolved on 10% NuPAGE Novex Bis-Tris gels, according to the manufacturer's specifications (Novex, San Diego, CA). All samples in these studies were denatured and reduced in SDS-PAGE buffer containing DTT. Proteins were transferred to 0.45-μm nitrocellulose membranes, blocked, and probed in 1× PBS, pH 7.4, 5% non-fat dry milk, 0.05% Tween-20, and 0.1% thymerosal. Membranes were then incubated in LumiGlo chemiluminescent substrate (KPL) and exposed to Kodak BioMax MS Film. Developed films were subjected to high resolution scanning for densitometry analysis. Quantification of band intensity was performed using National Institutes of Health ImageJ 1.41o software , and following the procedure outlined in , using TIFF files.

### Immunoprecipitation

Soluble GP1 was immunoprecipitated from 1 mL of transfected culture supernatants with 2 μg of anti-LASV GP1 mAb L52-74-7A and Protein G-Sepharose^® ^4B Fast Flow, using a Protein G Immunoprecipitation Kit (Sigma), according to the manufacturer's instructions. Immunoprecipitated proteins were denatured and reduced, separated by SDS-PAGE, and blotted as outlined above. GP1 was detected with biotinylated anti-LASV GP1 mAb L52-74-7A and ImmunoPure^® ^Streptavidin-HRP (Thermo Scientific).

### Cell proliferation assay

HEK-293T/17 cell cytotoxicity induced by metalloprotease inhibitors was monitored with a TACS™ MTT Cell Proliferation Assay (R&D Systems, Minneapolis, MN), according to manufacturer's instructions. The transfection procedure outlined above for 6-well plates was scaled down to a 96-well format, with each condition analyzed in triplicate. Data was plotted as mean absorbance at 570 nm, with standard deviation, and background correction at 650 nm.

### Immunofluorescence assay

HEK-293T/17 cells were seeded on Poly-D-Lysine-coated sterile glass cover slips (BD Biosciences, Walkersville, MD) in 24-well plates. Cells were transfected as outlined above. At 72 hours post-transfection cells were washed with cold HBSS and were either fixed in 2% methanol-free formaldehyde, or incubated in 1× PBS, pH 7.4/1% heat inactivated FBS/0.1% NaN_3 _for live cell staining. Fixed cells were incubated for 20 minutes in 50 mM NH_4_Cl to reduce background fluorescence, followed by permeabilization with 0.1% Triton-X 100 for 5 minutes, and blocking with 8% heat inactivated goat serum for one hour. Cells were stained with anti-LASV GP1 mAb L52-74-7A diluted to 2 μg/mL in 0.8% goat serum/0.1% Triton-X 100, for one hour. After washing, cells were incubated with Alexa Fluor 488 Goat F(ab')_2 _anti-mouse IgG (Molecular Probes/Invitrogen) diluted to 1 μg/mL in staining buffer containing 1 μM DAPI (Invitrogen), for 1 hour. Cover slips were mounted on ethanol washed glass slides with ProLong^® ^antifade reagent (Invitrogen). Reactions were cured for 48 hours prior to microscopy. Live cells were stained with the same primary, secondary antibodies and DAPI, for one hour each in 1× PBS, pH 7.4/1% heat inactivated FBS/0.1% NaN_3_, followed by mounting and curing, as described above. All reactions were performed at room temperature, and were washed three times with 1× DPBS between steps. Stained cells were imaged with a Zeiss Axioplan 2 fluorescent microscope equipped with an Intelligent Imaging Innovations CCD (Denver, CO), using a Plan-APOCHROMAT 63X/1,4 oil DIC objective.

### PNGase F, Endo H, and Neuraminidase assays

The glycosylation patterns in secreted GP1 generated from expression of LASV GPC and sGP1 were resolved by treatment with the glycosidases PNGase F, Endo H, and Neuraminidase. Immunoprecipitated GP1 from transiently transfected HEK-293T/17 cell supernatants were subjected to treatment with 500 NEB U of PNGase F, Endo H, or Neuraminidase for 1 hour using the reaction conditions suggested by the manufacturer (New England Biolabs). Control reactions were similarly processed except that enzymes were not added. Following incubation proteins were resolved by reducing SDS-PAGE, blotted, probed with biotinylated anti-LASV GP1 mAb L52-74-7A and Streptavidin-HRP, and developed as described above.

### Lectin-based Glycan differentiation assays

Glycosylation patterns on secreted GP1 were further characterized via binding of glycan-specific lectins using a DIG Glycan Differentiation Kit (Roche Applied Science, Mannheim, Germany), according to the manufacturer's instructions. Immunoprecipitated GP1 from expression of LASV GPC and sGP1 was resolved on SDS-PAGE, blotted onto nitrocellulose, and subjected to lectin binding assays.

### N-terminal protein sequencing

Immunoprecipitated GP1 protein was resolved by SDS-PAGE, blotted onto PVDF membranes, stained with Ponceau Blue and destained in water. N-terminal sequence determination of the 42 KDa protein was performed by Edman degradation and carried out in an Applied Biosystems Model 4949 CLC protein suquenator. Phenylthiohydantoin derivatives of amino acids were analyzed on-line with an Applied Biosystems Model 785A/140C/610A analyzer. All reagents and solvents were from Applied Biosystems.

### Statistical analyses

Statistical analysis of data was performed with GraphPad InStat, V3.06 (GraphPad Software, Inc., San Diego, CA), using Analysis of Variance (ANOVA).

## Competing interests

LMB and RFG are listed inventors, in addition to others, in a PCT application entitled "Soluble and Membrane-Anchored Forms of Lassa Virus Subunit Proteins", filed in April 2008.

## Authors' contributions

LMB contributed to the experimental design, engineered the expression systems, performed data analysis, and drafted the manuscript. RFG contributed to the experimental design and provided critical review of the manuscript.
